# Study on a Fault Diagnosis Method for Heterogeneous Chiller Units Based on Transfer Learning

**DOI:** 10.3390/e27101049

**Published:** 2025-10-09

**Authors:** Qiaolian Feng, Yongbao Liu, Yanfei Li, Guanghui Chang, Xiao Liang, Yongsheng Su, Gelin Cao

**Affiliations:** 1 College of Power Engineering, Naval University of Engineering, Wuhan 430030, China; 2 School of Mechatronics Engineering, Wuhan University of Technology, Wuhan 430070, China

**Keywords:** chiller unit fault diagnosis, transfer learning, dual-channel autoencoder, domain adversarial training, pseudo-labels, domain adaptation

## Abstract

As the core refrigeration equipment in cooling systems, the operational state of chiller units is crucial for ship support, equipment cooling, and mission stability. However, because of their sensitivity and the complexity of operating environments, obtaining large volumes of complete, fault-labeled data is difficult in practical engineering appli-cations. This limitation makes it challenging for traditional data-driven approaches to deliver accurate fault diagnoses. Furthermore, data collected from different devices or under varying operating conditions often differ significantly in both feature dimensions and distributions, i.e., data heterogeneity, which further complicates model transfer. To address these challenges, this study proposes a deep transfer learning–based fault di-agnosis method designed to leverage abundant knowledge from the source domain while adaptively learning features of the target domain. Given the persistent difficulties in collecting sufficient high-quality labeled fault data, traditional data-driven models continue to face restricted diagnostic performance on target equipment. At the same time, data heterogeneity across devices or operating conditions intensifies the challenge of cross-domain knowledge transfer. To overcome these issues, this study develops a heterogeneous transfer learning method that integrates a dual-channel autoencoder, domain adversarial training, and pseudo-label self-training. This combination enables precise small-sample knowledge transfer from the source to the target domain. Specifi-cally, the dual-channel autoencoder is first applied to align heterogeneous feature di-mensions. Then, a Gradient Reversal Layer (GRL) and a domain discriminator are in-troduced to extract domain-invariant features. In parallel, high-confidence pseu-do-labeled samples from the target domain are incorporated into joint training to im-prove generalization and robustness. Experimental results confirm that the method achieves high fault diagnosis accuracy in typical industrial application scenarios, ena-bling effective identification of common faults in various types of chiller units under conventional operating conditions, the proposed method achieves higher accuracy and F1-scores in multi-class fault diagnosis tasks compared with both traditional approaches and existing transfer learning methods. These findings provide a novel perspective for advancing the intelligent operation and maintenance of chiller units.

## 1. Introduction

Being a standard heat- and cold-source device, the chiller unit is a fundamental element of port energy and power systems and is commonly used in emergency situations, including command and control, ship support, and equipment cooling. Optimization of building energy systems has gained more and more research interest in recent years due to the so-called dual-carbon objective and the smart redesigning of energy systems [1]. It has been statistically demonstrated that the construction of energy system consumes over one-third of the world’s energy and almost 40% of direct and indirect carbon emissions [2], and heating, ventilation, and air-conditioning (HVAC) systems consume approximately 40% of total building energy use [3]. Chiller units are generally subjected to high loads, variable working conditions, and humid marine conditions in naval port stations and therefore are highly susceptible to performance degradation caused by condenser fouling [4], abnormal water flow, refrigerant leakage and other problems. Although these latent faults might not raise immediate alarm, they slowly diminish performance [5], and in extreme instances, lead to cooling failures in ship borne systems, hence compromising mission preparedness and supportability.

Different Fault Detection and Diagnosis (FDD) approaches to chiller units have been suggested to enhance equipment reliability and system assurance, typically divided into rule-based [6], model-based [7], and data-driven approaches [8]. The former two are strongly dependent on expert knowledge and system-specific expertise, and hence, their generalization in complex and changing conditions is constrained [9]. As sensing, artificial intelligence, and big data technologies have advanced, data-driven approaches have become popular in intelligent fault diagnosis [10]. Specifically, deep learning, due to its powerful feature extracting potential, has demonstrated significant benefits in chiller fault diagnosis [11]. As an example, Gao [12] created an SP-CNN model using data augmentation and convolutional neural networks to identify common types of faults with high precision. In the same way, Lu et al. [13] proposed a better variational autoencoder with a co-training mechanism, which improves the data quality and fault detection performance.

In spite of these developments, the practical use of deep learning in engineering remains a challenge. Deep learning models typically need massive amounts of high-quality labeled data to be trained. In highly reliable industrial applications like the chiller units in the naval port, however, the sensitivity of the operations, the infrequence of fault occurrences, and the complexity of data collection complicate the process of obtaining adequate labelled fault data. This is of particular concern in cross-equipment applications, where labelled samples in the target domain are very few. In addition, the majority of current research is confined to individual devices or homogeneous data sets and lacks the generalization required across devices and operating conditions. The traditional methods tend to fail when transferring a model between a device (source domain) and another (target domain) with different feature dimensions and data distributions (heterogeneity), and where the target domain has only very few, or none, labeled samples [14]. Therefore, correct diagnosis of chiller faults in cross-equipment and small-sample conditions has become an urgent problem. Transfer learning offers an encouraging solution to the two issues of data scarcity and inter-domain heterogeneity [15].

Transfer learning seeks to apply knowledge acquired in prior task domains to new tasks [16]. By integrating labeled data from the source domain with unlabeled data from the target domain, it generates a classifier for the target domain, thereby effectively addressing distributional differences across datasets. Wei et al. [17] proposed an end-to-end multilayer support vector machine (ML-SVM) architecture that combines transfer learning with an improved oversampling strategy. This approach mitigated data imbalance, reduced noise, and lessened reliance on handcrafted features in multi-class fault diagnosis of mechanical systems. Similarly, Na et al. [18] introduced a heterogeneous transfer learning method for railway track fault prediction. Their approach reduced the dimensionality of both source- and target-domain features using autoencoders and subsequently constructed a heterogeneous transfer learning network, achieving 99% accuracy on the test dataset.

The literature discussed above suggests that transfer learning has found extensive application in industrial fault diagnosis. But when the fault data of the target equipment are highly sparse or completely unlabeled, source-domain supervision or feature alignment alone cannot be used to achieve high-accuracy diagnosis [19]. Pseudo-labeling has become an important method of unlabeled transfer learning in recent years. Its fundamental idea is to apply a trained model to the label prediction of samples in the target domain and use high-confidence predictions as pseudo-labels in the training process, which enhances adaptability and generalization to the target domain. Wei et al. [20] suggested a semi-supervised domain generalization approach that relies on a domain knowledge-directed pseudo-label generation framework, which generated high-quality labels and improved the strength of cross-domain generalization. On the same note, Chen et al. [21] introduced the Multi-stage Alignment of Multiple Source Subdomains Adaptation (MAMSA) approach that used pseudo-labels generated by the classifier to guide and align it, greatly enhancing diagnostic performance in cross-condition settings. An interpretable domain adaptation transformer has been proposed that leverages the global modeling capabilities of the Transformer architecture and domain adaptation techniques to effectively learn fault features across different operating conditions and machines [22]. This method provides model interpretability through an ensemble attention weight mechanism and takes raw vibration signals as input, demonstrating its effectiveness in various transfer diagnostic tasks. A deep transfer learning method [23] has been proposed, which effectively improves the accuracy of cross domain gear fault diagnosis through variational mode decomposition and pre-processing of Gramian angle field, improved residual attention network, and staged transfer training strategy, especially in the case of limited labeled data and significant differences between source and target domains. A transfer learning method [24] based on improved elastic network was proposed, combined with long short-term memory network (LSTM), to achieve high-precision bearing fault diagnosis under variable load conditions. This method suppresses overfitting and improves training efficiency through elastic networks, requiring only a small amount of target domain data fine-tuning to significantly outperform traditional LSTM, GRU, and Bi LSTM models, demonstrating stronger generalization ability and effectiveness. The articles [25,26,27,28] reflect two major trends in the field: first, the evolution from models heavily reliant on large amounts of labeled data toward advanced AI models capable of few-shot learning, strong generalization, and privacy preservation (such as domain generalization and federated learning); second, the systematic review and summarization of core tasks in predictive maintenance (e.g., RUL prediction) and key technologies (e.g., health indicators) to further advance the discipline.

Based on these results, this paper concentrates on the chiller unit of a naval port station, where multi-class fault diagnosis scenarios are built and a transfer learning approach is proposed, which combines a domain adversarial training mechanism with a Gradient Reversal Layer (GRL) and a pseudo-label self-training strategy. The proposed method significantly enhances the cross-equipment diagnostic potential and resilience of chiller units, even under the circumstances of having only a few fault labels in the target domain, by incorporating heterogeneous feature unification at the data level, source-domain model initialization, domain adversarial training, pseudo-label generation, and joint training. This paper presents a novel framework for few-shot cross-equipment diagnosis of military-port chillers by innovatively combining a dual-channel sparse autoencoder with domain adversarial training and pseudo-label learning, which collaboratively boosts generalization via feature alignment and domain adaptation.

## 2. Methodology

### 2.1. Overall Structure of the Heterogeneous Transfer Learning Network

This study proposes a heterogeneous transfer learning framework for chiller units, designed for small-sample fault prediction tasks. The framework’s primary objective is to address the scarcity of target-domain data through cross-domain knowledge transfer. Given the substantial differences between the data spaces of the source and target domains, a heterogeneous transfer learning network is developed. This network integrates heterogeneous feature encoding techniques with a domain adversarial network, thereby overcoming challenges caused by variations in feature dimensions and distributions across domains. To further enhance diagnostic accuracy under limited samples, pseudo-label learning is incorporated.

**Figure 1:**
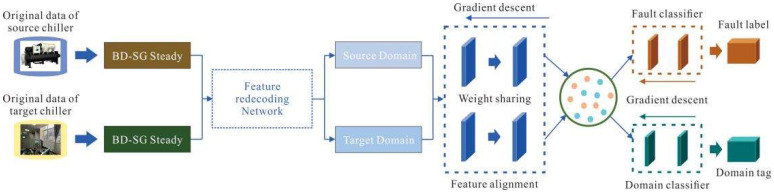
Overall Framework of the Transfer Learning Network.

In this study, the Ra-1043 public dataset of chiller units is adopted as the source domain. The collected chiller unit data are first preprocessed through filtering and steady-state feature extraction. The steady-state data of varying dimensions obtained from both the source and target domains are then used as inputs to construct and train a heterogeneous feature encoding network, aligning feature dimensions between the two domains. A domain adversarial neural network is subsequently employed to build the diagnostic model, which is trained to accomplish the fault diagnosis task. Finally, a small portion of target-domain data is used to fine-tune the model, enabling improved performance in target-domain fault prediction. The overall framework is illustrated in Figure 1.

The heterogeneous transfer learning network illustrated in Figure 1 leverages the abundant public chiller unit dataset to accelerate the training of the target-domain fault prediction model. By utilizing source-domain data, it substantially reduces the amount of target-domain data required during training. The following sections provide a detailed description of the proposed heterogeneous transfer learning network.

### 2.2. Dual-Channel Feature Re-Encoding Network

In cross-equipment fault diagnosis of chiller units, dual heterogeneity exists between the source domain (high-dimensional sensors, 60+ dimensions) and the target domain (low-dimensional sensors, 20+ dimensions). The source domain contains high sensor redundancy, including complex parameters such as oil temperature and oil pressure, whereas the target domain is limited to basic parameters such as temperature, pressure, and power. These differences in the physical meaning of features lead to $p_{s}(X)\neq p_{t}(X)$, and forcing alignment through a single autoencoder often results in information loss. To address this challenge, a dual-branch autoencoder is designed, in which heterogeneous feature unification is achieved through independent encoding paths. An autoencoder, composed of an encoder and a decoder, is an unsupervised neural network widely applied in feature extraction tasks.

In this study, a left–right symmetrical dual-channel sparse autoencoder is adopted. It independently encodes and reconstructs the source-domain input $X_{s}\in R^{d_{s}}$ and the target-domain input $X_{t}\in R^{d_{t}}$, while applying sparsity constraints on the hidden layer to enhance both the discriminability and sparsity of feature representations. This design facilitates subsequent cross-domain alignment and transfer.

**Figure 2:**
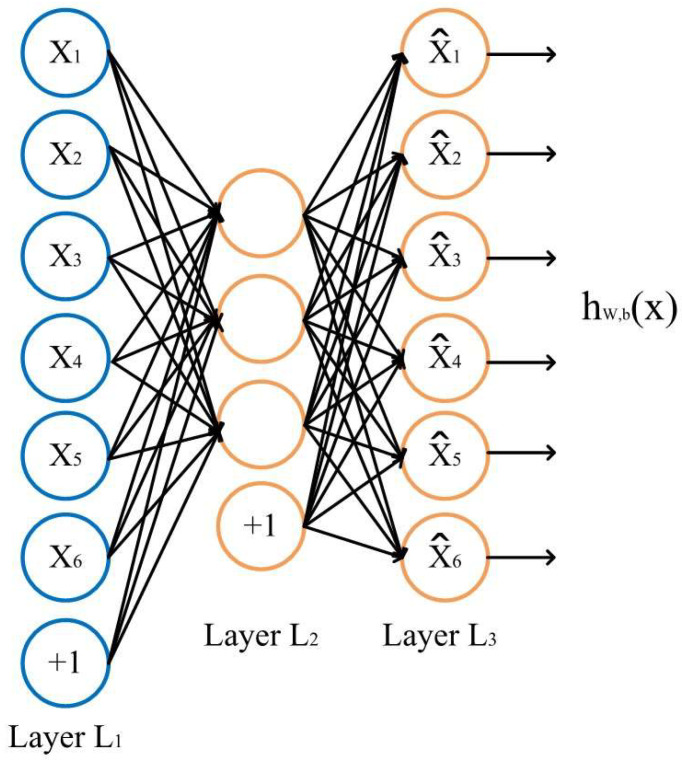
Structure of the Sparse Autoencoder.

The structure of the sparse autoencoder is illustrated in Figure 2.

The source and target domains each employ their own encoder–decoder structure.

$$z_{s}=E_{s}\left(X_{s};\theta_{s}\right),\;{\hat{x}}_{s}=D_{s}\left(z_{s};\emptyset_{s}\right)$$

$$z_{t}=E_{t}\left(X_{t};\theta_{t}\right),\;{\hat{x}}_{t}=D_{t}\left(z_{t};\emptyset_{t}\right)$$

By designing encoders $E_{s}$ and $E_{t}$, the method generates latent representations $z_{s},z_{t}\epsilon R^{L}$ (where L is the unified latent dimension), thereby mapping the different input spaces into a common latent space. The conventional reconstruction loss is defined as the Mean Squared Error (MSE):

$$L_{\mathrm{rec}}=∥x_{s}-{\hat{x}}_{s}{∥}_{2}^{2}+∥x_{t}-{\hat{x}}_{t}{∥}_{2}^{2}$$

To encourage the hidden layer to generate sparse representations, a $Kullback-Leibler\;\left(KL\right)$ divergence penalty term is introduced between the average activation ${\hat{p}}_{j}$ of each hidden unit and the target sparsity rate ρ. For a batch of m samples, the average activation of the *j*-th hidden unit is defined as:

$${\hat{\rho }}_{j}=\frac{1}{m}\sum_{i=1}^{m}z_{j}^{\left(i\right)}$$

The sparsity regularization term is defined as:

$$L_{sparse}=\sum_{j=1}^{L}\;KL(\rho ∥{\hat{\rho }}_{j})=\sum_{j=1}^{L}\;\left(\rho log\frac{\rho }{{\hat{\rho }}_{j}}+(1-\rho )log\frac{1-\rho }{1-{\hat{\rho }}_{j}}\right)$$

The total loss of the dual-channel sparse autoencoder is expressed as:

$$L_{sae}=L_{rec}+\beta L_{sparse}$$

where β>0  controls the weight of the sparsity term.

The sparsity constraint suppresses redundant feature activations, promoting the learning of sparse bases that are both more discriminative and transferable. By independently learning domain-specific representations through the left and right channels and mapping them into a unified *L*-dimensional latent space, the method preserves the distinctive characteristics of each domain while simultaneously providing aligned data for subsequent domain adversarial training.

### 2.3. Domain Adversarial Neural Network

#### 2.3.1. Fundamental Theory of Domain Adaptation

The core objective of cross-domain learning is to enable knowledge transfer between the source domain $D_{s}={\left\{\left(x_{s}^{i},y_{s}^{i}\right)\right\}}_{i=1}^{N_{s}}$ and the target domain $D_{t}=\{x_{t}^{j}{\}}_{j=1}^{N_{t}}$, where $X_{s}\in R^{d_{s}}$ and $X_{t}\in R^{d_{t}}$ share the same label space but differ in data distributions, i.e., $P_{s}(x,y)\neq P_{t}(x,y)$. Domain adaptation (aimsDA) seeks to ensure, through feature transformation and model training, that the learned feature distributions satisfy:

$$P_{s}(f(x_{s}))\approx P_{t}(f(x_{t}))$$

Thus, a classifier trained on the source domain can retain strong generalization performance when applied to the target domain. Domain adaptation methods are generally classified into three categories: (1) Feature transformation methods, which explicitly align feature distributions using mapping functions such as MMD or CORAL. (2) Adversarial training methods, which introduce a domain discriminator and a gradient reversal mechanism, enabling the feature extractor to generate domain-invariant features. (3) Hybrid methods, which combine distribution distance metrics with adversarial optimization to further enhance alignment effectiveness.

The domain adversarial neural network (DANN) adopted in this study falls into the second category. Its main advantage is that it avoids the need for an explicit definition of distribution distance and can be jointly optimized end-to-end with deep feature extraction.

#### 2.3.2. Structure of the Domain Adversarial Neural Network

The core concept of DANN is the introduction of a GRL between the feature extractor and the domain discriminator. During forward propagation, the features remain unchanged; however, in backpropagation, the gradient signs are reversed, thereby enabling adversarial training between the feature extractor and the domain discriminator.

**Figure 3:**
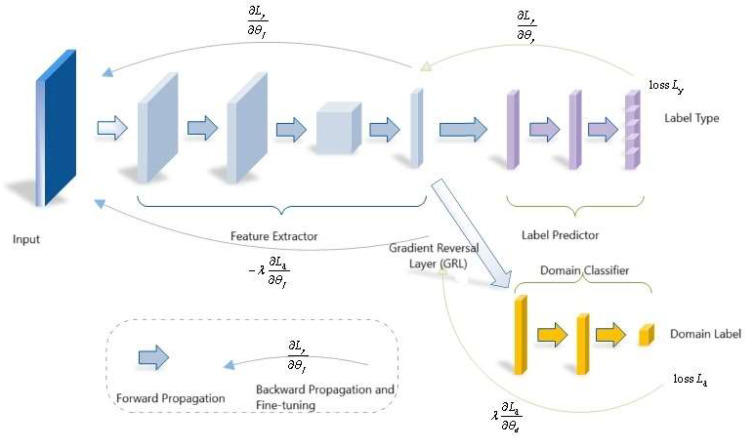
Domain-Adversarial Neural Network Structure.

To address the challenges in cross-domain fault diagnosis of chiller units, where the source and target domains differ in feature dimensions and exhibit significant distributional discrepancies, this study incorporates a dual-channel sparse autoencoder (SAE) into the feature extraction stage of the classical DANN framework to achieve heterogeneous feature mapping. Combined with a CNN and domain adversarial optimization, the framework supports end-to-end training, as shown in Figure 3.

The proposed network consists of three components: (1) Dual-channel sparse autoencoder (independently SAE): Independently encodes the input features of the source and target domains, mapping them into unified latent dimension, while introducing sparsity L. Sparsity constraints are introduced in the latent space to enhance feature discriminability and suppress redundant information. (2) Shared Feature Extractor CNN feature extractor and label classifier: The latent features output by the SAE are further processed by a shared CNN to extract deeper local patterns and temporal features.

The label classifier C(⋅) receives the *CNN* output, predicts labels for source-domain samples, and is optimized using cross-entropy loss:

$$L_{cls}=-\frac{1}{N_{s}}\sum_{i=1}^{N_{s}}\;\sum_{k=1}^{K}\;y_{s,k}^{i}logC_{k}(F(x_{s}^{i}))$$

GRL and Domain Discriminator: The domain discriminator is a binary classification network designed to determine whether the input features originate from the source domain or the target domain. During adversarial training, the domain discriminator and the feature extractor (i.e., the source-domain compressor and target-domain expander) engage in a minimax game. The discriminator seeks to correctly classify the origin of features, while the feature extractor, through the *GRL*, learns to generate domain-invariant feature representations that “fool” the discriminator. This adversarial process compels the model to extract domain-independent general features, thereby achieving domain alignment. The optimization objective of the domain discriminator is expressed as:

$$L_{adv}=-E_{z_{s}}[logD(z_{s})]-E_{z_{t}}[log(1-D(z_{t}))]$$

where $z_{s}\;and\;z_{t}$ denote the shared features of the source domain and the target domain, respectively.

The joint optimization function is:

$$\underset{F,C}{min}\;\underset{D}{max}\;L_{sae}+\lambda_{cls}L_{cls}-\lambda_{adv}L_{adv}$$

In this function, where $\lambda_{cls}$, and $\lambda_{adv}$ denote the loss weights for each task, respectively.

## 3. Dataset Description

In this section, we describe the dataset used to validate the proposed transfer learning method. Experimental studies were conducted on different chiller units under various operating conditions, and both source-domain and target-domain data were collected. The source-domain data were obtained from the ASHRAE-1043 project, while the target-domain data were gathered from a research project on a specific type of screw chiller. The following subsections provide a detailed description of the chiller systems and data collection procedures. In addition, the considered fault types are introduced, and the features employed in this study are summarized.

### 3.1. Source-Domain Chiller Unit

The source-domain data were obtained from the operational dataset of the chiller unit in the ASHRAE-1043 project. The system consisted of a 316 kW indoor centrifugal chiller using tetrafluoroethane (R-134a) as the refrigerant, designed to investigate six typical chiller faults with varying severity levels (SL). Chiller faults can be primarily categorized into failure-mode faults and degradation-mode faults. Most failure faults can be easily detected with simple albeit sometimes expensive equipment. Degradation faults, on the other hand, generally lead to a loss of performance, but are otherwise not easily detected (since the chiller is often still operational) and cannot normally be detected with a single sensor. Consequently, the degradation faults are shown in Table 1. Cumulatively, they account for 42% of the service calls. Therefore, these degradation-mode faults were selected as the research focus. Both the evaporator and condenser employed shell-and-tube configurations and were installed as water-cooled flooded-type heat exchangers. To simulate different operating conditions, the original system was modified by adding auxiliary water and steam circuits.

The system was first operated in a fault-free state, after which faults were artificially introduced under laboratory conditions at nearly constant ambient temperatures. The severity of each fault was progressively increased across the test series. A set of predetermined test sequences was defined using the evaporator outlet water temperature, condenser inlet water temperature, and calculated evaporator cooling rate as control variables. Based on these sequences, 27 operating states were established. Each state was continuous, with every fault reaching a steady-state condition before transitioning to the next.

The following is the fault list 1 used in this study as in Table 1:

### 3.2. Target-Domain Chiller Unit

For the target-domain dataset, the system consisted of two parts: the experimental test bench and the data acquisition system. The main equipment of the cooling station test bench was a screw chiller unit operating under a return-water temperature control strategy. The chiller unit comprised a compressor, condenser, expansion valve, evaporator, piping accessories, and an electrical control cabinet, all mounted on a common base frame. The data acquisition system was connected to the test bench through sensors such as temperature and pressure transducers. It included both hardware and software components: the hardware comprised data acquisition devices and sensors for temperature, humidity, pressure, flow rate, and electrical parameters, while the software supported data visualization, processing, and storage.

**Figure 4:**
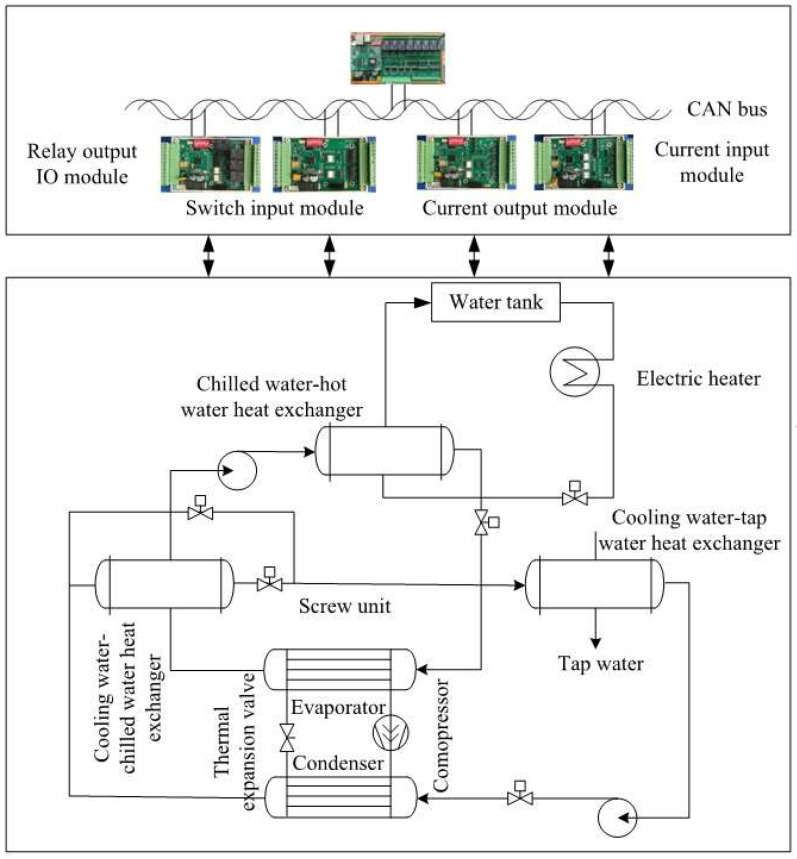
Schematic Diagram of the Cooling Station Experimental Test Bench.

The experimental system and measurement point layout are shown in Figure 4. As illustrated, the system incorporated not only load control but also fault simulation devices, such as a bypass valve between the compressor suction and discharge pipelines, adjustment valves on the refrigerant circuit, and regulating valves for both cooling and chilled water. Parameter signals from all measurement points were transmitted to the PC host computer via a data acquisition card, enabling continuous sampling, real-time display, and data recording. The performance parameters under rated conditions are shown in Table 2.

**Figure 5:**
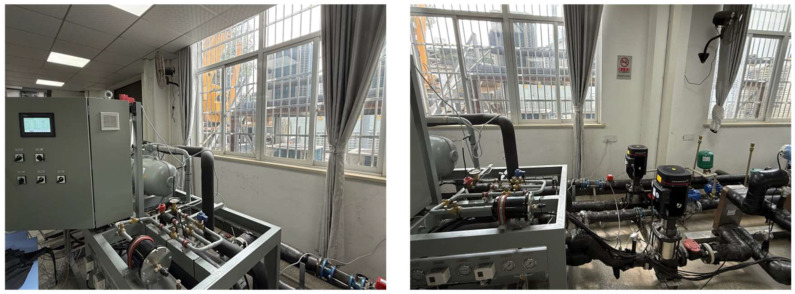
Physical Diagram of the Target Chiller Unit.

Figure 5 presents a photograph of the actual device. Data collection for the target chiller unit was performed under rated working conditions. The outlet temperature of the chilled water was controlled at 7 °C, while the return temperature of the cooling water was maintained at 20 °C. The compressor capacity was adjusted automatically. After the unit was powered on, the control system executed a self-check, and once no faults were detected, the operating mode was switched to “automatic.” The desired target temperature was then set on the touchscreen, and pressing the automatic start button initiated the reduced-voltage start logic: the compressor contactor engaged, the operation indicator illuminated, and the unit regulated its capacity according to the programmed control logic, using the chilled-water outlet temperature as the reference. The operating status and parameters of the unit could be monitored in real time on the touchscreen.

Reduced condenser water flow rate: On the control screen, the opening of the motorized two-way cooling-water valve (100% indicating fully open) was adjusted. The cooling-water flow rate was monitored and reduced to 90%, 80%, and 70% of the nominal 20 m^3^/h. After each adjustment, the unit was allowed to stabilize before data variations were observed and recorded.

Reduced evaporator water flow rate: Similarly, the opening of the motorized two-way chilled-water valve (100% indicating fully open) was adjusted. The chilled-water flow rate was monitored and reduced to 90%, 80%, and 70% of the nominal 12 m^3^/h. After each adjustment, the unit was stabilized before recording data variations.

**Figure 6:**
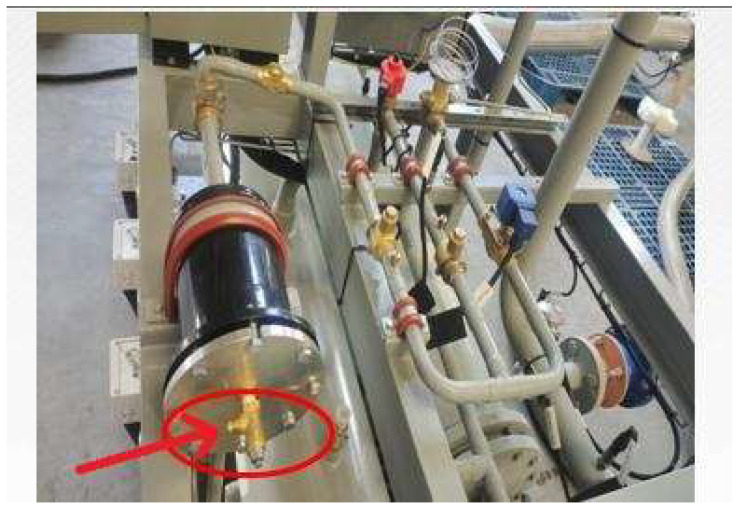
Proportional Charging of Refrigerant through the Liquid Charging Valve.

Excessive refrigerant charge: At the location indicated in Figure 6, additional R134a refrigerant was charged in increments of 3 kg (10%), 6 kg (20%), and 9 kg (30%). Following each overcharge, the unit was allowed to stabilize, after which data variations were observed and recorded.

Insufficient refrigerant charge: After the unit was fully evacuated, R134a refrigerant was recharged at the location shown in Figure 6 in increments of 18 kg (60%), 22.5 kg (80%), and 27 kg (90%) of the nominal charge. Following each incremental charge, the unit was allowed to stabilize before data variations were observed and recorded.

Presence of non-condensable gases: At the location indicated in Figure 4, nitrogen was injected into the system in increments of 3 kg (10% of the refrigerant charge) and 6 kg (20%). After each injection, the unit was allowed to operate until stabilization, after which data variations were observed and recorded. Table 3 presents the fault simulation table:

The variables collected by the sensors are listed in Table 4:

### 3.3. Data Processing

Since chiller unit operation typically involves startup, steady-state, and shutdown stages, the raw data exhibit strong non-stationarity. To improve the stability and robustness of the diagnostic model, a steady-state detector was developed using the Savitzky–Golay (SG) filter in combination with the backward difference (BD) method to identify and extract representative steady-state operating segments.

As a front-end preprocessing module, the SG filter applies local polynomial fitting for signal smoothing. Specifically, a second-order polynomial (polyorder = 2) is fitted within a sliding window of 15 points (equivalent to 1.5 min of data) using the least squares method, and the central point of the window is replaced by the fitted value. The selection of window length and polynomial order balances noise suppression against feature preservation: larger window lengths improve smoothing but risk diminishing abrupt features (e.g., temperature steps induced by faults), whereas low-order polynomials (order 2–3) maintain the overall signal shape while avoiding overfitting.

**Figure 7:**
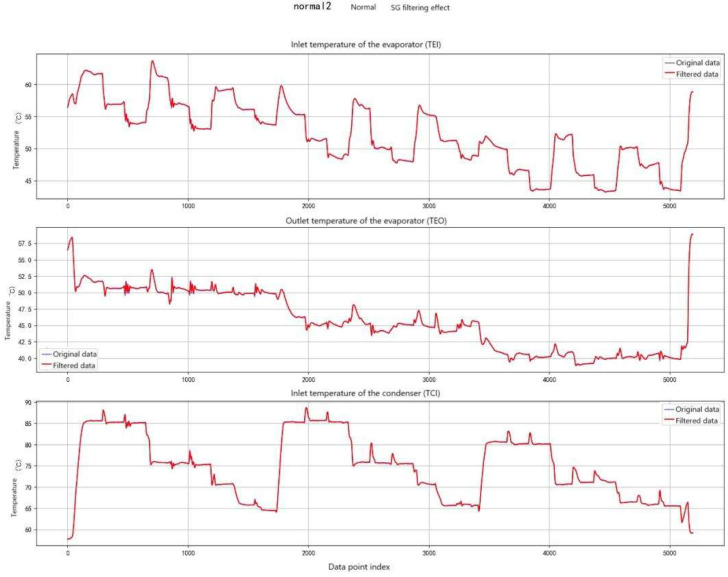
SG Filtering Diagram.

Compared with traditional moving-average filtering, the SG filter exhibits superior shape-preserving performance. It retains peak and edge characteristics (e.g., early fault fluctuations of ±0.5 °C) and avoids the feature loss caused by excessive smoothing. The results of SG filtering are illustrated in Figure 7.

BD Method: A geometrically weighted steady-state detection algorithm was employed, with its core principle relying on statistical feature analysis within a sliding window. The window length was set to 180 s (tau_ss), and the sampling interval was 10 s (delta_t). To emphasize recent observations, an exponential decay weight was applied, with the weighting coefficient defined as β=τ_ss/(τ_ss+Δt)≈0.947).

**Figure 8:**
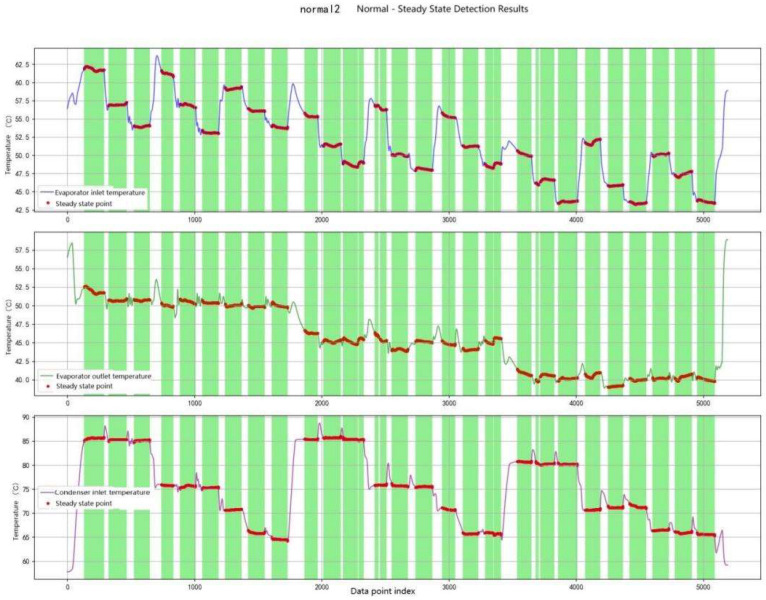
BD Steady-State Detection Diagram.

Within each sliding window, the weighted standard deviations of the evaporator inlet temperature (TEI), evaporator outlet temperature (TEO), and condenser inlet temperature (TCI) were calculated. When the standard deviations of all three parameters simultaneously fell below the threshold of 0.1 °C, the system was considered to have reached a steady-state condition. The extracted steady-state data were subsequently used as inputs for feature learning and classification. The results of BD-based steady-state detection are presented in Figure 8.

Table 5 presents the processed datasets of the source-domain and target-domain chiller units, where each type of fault data is divided into three categories.

## 4. Model Training and Detailed Settings

### 4.1. Training Procedure

This study proposes a dual-stage joint training framework to address the challenges of scarce labeled data in the target domain (screw chiller units) and cross-domain distribution discrepancies in fault diagnosis for naval vessel chiller systems. The framework combines pseudo-label self-training and domain adversarial training to achieve effective knowledge transfer from the label-rich source domain to the target domain. The overall training process is illustrated in the figure below, which can be understood from three aspects:

Data Input and Feature Mapping

Data Sources: The model simultaneously receives two types of data:

Source Domain Data: From the public ASHRAE-1043 dataset, containing abundant fault samples and corresponding labels.

Target Domain Data: Operational data from the target naval vessel screw chiller units, with only limited or no labels.

Feature Preprocessing and Unification: Source and target domain data are preprocessed separately using sparse autoencoders (SAE_1 and SAE_2). This step aims to learn sparse representations of the data, filter out noise, and project heterogeneous data into a unified latent feature space, laying the foundation for subsequent domain alignment.

Core Mechanism: Dual-Stage Joint Training

The core of the framework lies in the synergistic iteration of pseudo-label generation and domain adversarial training.

Stage 1: Pseudo-Label Self-Training

The model first trains a shared CNN feature extractor and classifier using labeled source domain data to establish preliminary fault diagnosis capability. This model is then applied to the target domain data to generate high-confidence pseudo-labels. These high-quality target domain samples with pseudo-labels are incorporated into the training set to gradually guide the model in adapting to the target domain characteristics.

Stage 2: Domain Adversarial Training

To achieve more thorough domain-invariant feature learning, the framework introduces a Gradient Reversal Layer (GRL) and a domain discriminator. The shared CNN feature extractor aims to generate features that can deceive the domain discriminator, making it unable to distinguish whether the features originate from the source or target domain. The domain discriminator, in turn, strives to correctly identify the feature sources. Through this adversarial process, the feature extractor is compelled to learn deep features that are domain-agnostic and highly generalizable.

Note: The two stages are not sequential but jointly conducted and mutually reinforcing. The quality of pseudo-labels improves as feature domain invariance enhances, while more accurate target domain pseudo-labels help the domain discriminator better model distributions, further improving domain alignment.

Model Optimization and Output

Optimization Strategy: The Adam optimizer combined with a StepLR learning rate scheduler is used to jointly optimize all model parameters. The optimization objective simultaneously minimizes the classification error (based on source domain true labels and target domain pseudo-labels) and the domain discrimination error.

Model Output: After training, a fault diagnosis model applicable to the target naval vessel screw chiller units is obtained.

Performance Evaluation: The final model is comprehensively evaluated on an independent test set using metrics such as Accuracy, F1-score, and Confusion Matrix.

**Figure 9:**
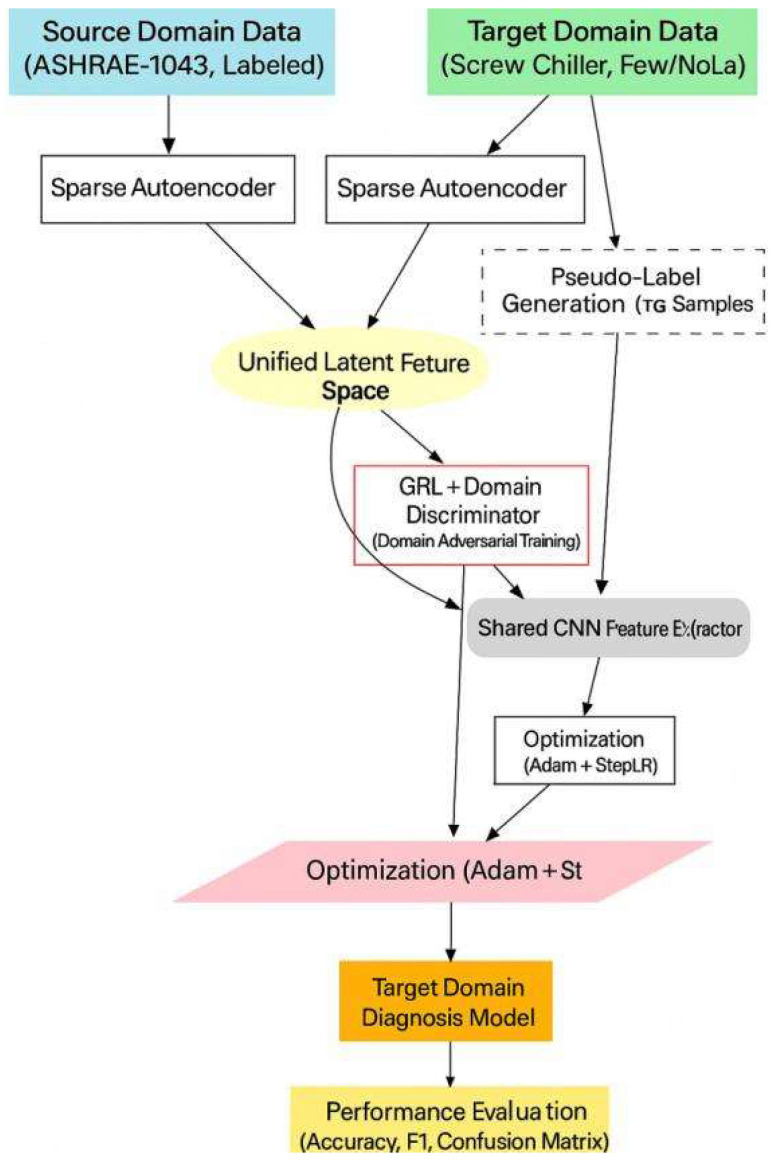
Diagram of the Two-Stage Joint Training Process.

The training flowchart is as follows in Figure 9:

### 4.2. Optimizer and Learning Rate Strategy

Optimizer: Adam, with an initial learning rate of 1 × 10^−4^, β1 = 0.9, β2 = 0.999;

Learning rate decay strategy: StepLR, decaying to 0.5 of the original rate every 20 epochs;

Weight decay coefficient: 0.0005;

Batch size: 64.

### 4.3. Parameter Settings

Hyperparameter settings have a significant impact on model performance. Based on preliminary experimental validation and common configurations in the field, the following key hyperparameter values were determined: the latent dimension was set to 64 to balance feature representation capability and computational complexity; the sparsity rate ρ was set to 0.05, and a sparsity penalty coefficient β = 0.1 was introduced to promote the learning of more discriminative sparse feature representations. In the loss function, the classification loss weight λ_cls was set to 1.0 as the primary supervisory signal for task optimization, while the adversarial loss weight λ_adv was set to 0.1 to enhance the model’s generalization ability and robustness. The total number of training epochs was set to 100 to ensure sufficient model convergence while avoiding overfitting. All hyperparameters were initially determined through grid search and cross-validation to ensure the reliability and reproducibility of the experimental results.

### 4.4. Model Implementation Details

During the model implementation process, to ensure experimental consistency and reproducibility, this study meticulously designed the data processing, regularization strategies, and evaluation methods. The specific details are as follows:

All input features were first subjected to normalization preprocessing. The Min-Max scaling method was employed to linearly transform the raw data into the [0, 1] interval, thereby eliminating the impact of feature scale differences on model training stability. After model training was completed, the target domain test set was used for performance evaluation. In addition to classification accuracy, the Macro-average F1-Score was adopted to provide a more comprehensive assessment of the model’s performance across different categories, which is particularly suitable for scenarios with imbalanced class distributions. Simultaneously, a Confusion Matrix was plotted to visually analyze the types of classification errors, facilitating an in-depth investigation of the model’s recognition capability and the sources of bias in practical scenarios.

## 5. Experimental Results

### 5.1. Evaluation Metrics

In evaluating model performance, this study selected multidimensional quantitative metrics to comprehensively reflect model characteristics: Accuracy was used as the fundamental metric to measure the overall classification correctness; the F1-score, by harmonizing precision and recall, effectively mitigated evaluation bias caused by data imbalance; the Confusion Matrix visually presented classification details across categories, revealing misclassification patterns for specific classes. Together, these three metrics not only capture overall performance but also help identify directions for local optimization.

### 5.2. Experimental Results and Analysis

#### 5.2.1. Training Loss

The training loss experiment systematically analyzed the loss dynamics during source-domain pretraining, adversarial training, semi-supervised learning, and fine-tuning stages, thereby comprehensively validating the effectiveness of the proposed phased domain adaptation strategy.

**Figure 10:**
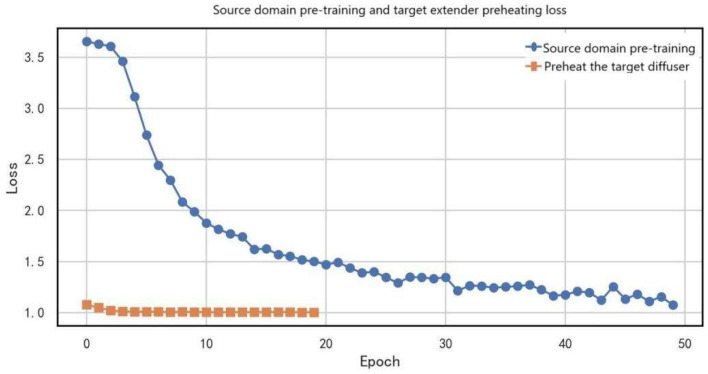
Loss variation curves in source-domain pretraining and target expander warm-up phases.

Figure 10 illustrates the loss variation curves during the source-domain pretraining and target expander warm-up phases. The source-domain pretraining loss (blue curve) significantly decreased from the initial 3.7 to 1.1 (a 70.3% reduction), showing a typical exponential decay trend, which indicates that the model effectively learned fault feature representations from the source domain. Notably, the target expander warm-up loss (orange curve) remained stably around 1.0 (with fluctuations within ±0.15). This low and stable loss suggests a high compatibility between the target and source domain feature distributions, where the target expander required only shallow feature transformations to achieve domain adaptation. The comparison of the two curves demonstrates the effectiveness of the staged training strategy adopted in this study: source-domain pretraining first completed deep feature extraction, while the target expander achieved domain alignment initialization at minimal computational cost, thereby laying a solid foundation for subsequent adversarial training.

**Figure 11:**
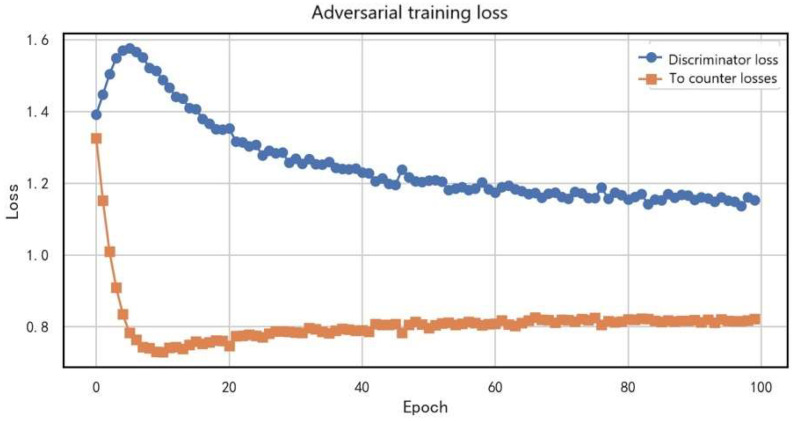
Dynamic relationship between discriminator loss and adversarial loss during adversarial training.

Figure 11 shows the dynamic relationship between discriminator loss and adversarial loss during adversarial training. The discriminator loss (blue curve) decreased significantly from the initial 1.6 to 0.6 (a 62.5% reduction), following a trend of rapid decline at the beginning and gradual stabilization later. This indicates that the discriminator quickly improved its domain discrimination ability in the early stage and then progressively reached stability. Notably, the adversarial loss (orange curve) remained consistently around 0.8 (with fluctuations within ±0.05). This stability suggests that the generator continuously produced domain-invariant feature representations throughout training. The comparison of the two curves reveals a typical adversarial training equilibrium: the steady decline in discriminator loss reflects its improving discriminative power, while the stable adversarial loss demonstrates the generator’s ability to effectively deceive the discriminator, enabling the two modules to reach dynamic balance. This loss variation pattern confirms the effectiveness of the Gradient Reversal Layer (GRL) and adversarial training mechanism adopted in this study, establishing a solid foundation for subsequent domain adaptation tasks.

**Figure 12:**
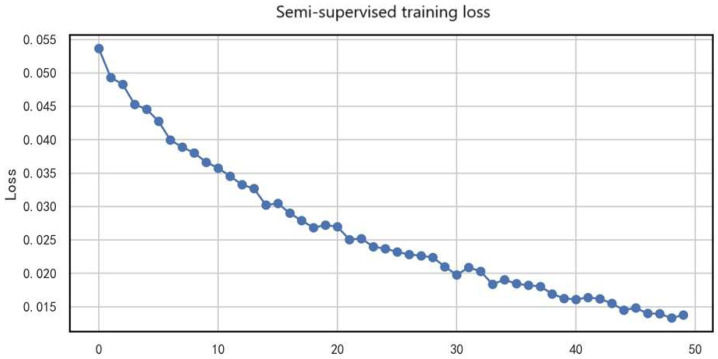
Loss values during semi-supervised training.

Figure 12 illustrates the variation of loss values with training epochs during semi-supervised training. The loss decreased steadily from the initial 0.055 to 0.015 (a 72.7% reduction), showing a clear convergence trend. Specifically, in the first 10 epochs, the loss dropped rapidly (from 0.055 to 0.030), indicating that the model quickly learned effective feature representations; during epochs 10–30, the decline slowed (from 0.030 to 0.020), suggesting that the model entered a stable optimization phase; finally, in epochs 30–50, the loss stabilized around 0.015, validating the effectiveness of the semi-supervised training strategy.

This convergence behavior indicates that the quality of pseudo-labels improved continuously during training; effective utilization of unlabeled data enhanced the model’s generalization ability; and no significant overfitting occurred.

The smooth decline of the loss curve demonstrates that the consistency regularization and confidence filtering mechanisms employed in this study effectively stabilized the semi-supervised learning process.

**Figure 13:**
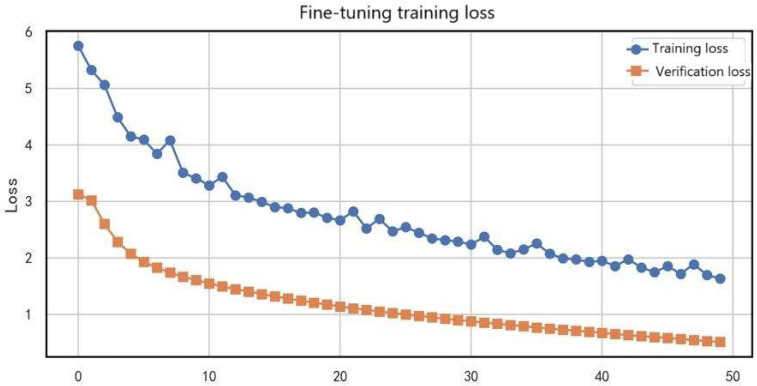
Training and validation loss curves during the fine-tuning phase.

Figure 13 presents the variation of training loss and validation loss during the fine-tuning phase. The training loss (blue curve) dropped rapidly from an initial value of 5.8 to around 0.3 (a 94.8% reduction), reaching convergence after about 40 epochs, indicating that the model effectively learned target-domain features. The validation loss (orange curve) simultaneously decreased from 4.2 to 0.6 (an 85.7% reduction), following a similar trend to the training loss, which suggests strong generalization capability. Notably, the two curves remained closely aligned (maximum gap < 0.5) without significant divergence, confirming that no overfitting occurred during fine-tuning. This convergence pattern demonstrates that the applied learning rate decay strategy (0.1× every 10 epochs) and weight regularization (L2 = 0.01) effectively stabilized the optimization process, enabling the model to achieve stable convergence within 50 epochs and providing a reliable guarantee for practical applications.

In summary, the exponential decay of source-domain pretraining loss (70.3% reduction) and the stability of the target expander loss (fluctuation ±0.15) confirmed the synergy between feature extraction and domain adaptation. The equilibrium observed during adversarial training—discriminator loss reduction of 62.5% with adversarial loss stability (fluctuation ±0.05)—revealed the mechanism by which the Gradient Reversal Layer achieved domain-invariant features. Furthermore, the 72.7% loss reduction in semi-supervised training, together with the synchronized convergence of training and validation losses during fine-tuning (gap < 0.5), further validated the role of pseudo-label enhancement and regularization strategies in improving generalization.

#### 5.2.2. Ablation Study

An ablation study was conducted to verify the effectiveness of three key components in the DDPN model: the attention mechanism, adversarial training, and semi-supervised learning. Four configurations were designed as follows:

Full model: included the attention mechanism, adversarial training, and semi-supervised learning.

Without attention mechanism: removed both channel attention and spatial attention modules.

Without adversarial training: removed the domain discriminator and Gradient Reversal Layer.

Without semi-supervised learning: removed the semi-supervised learning stage.

**Figure 14:**
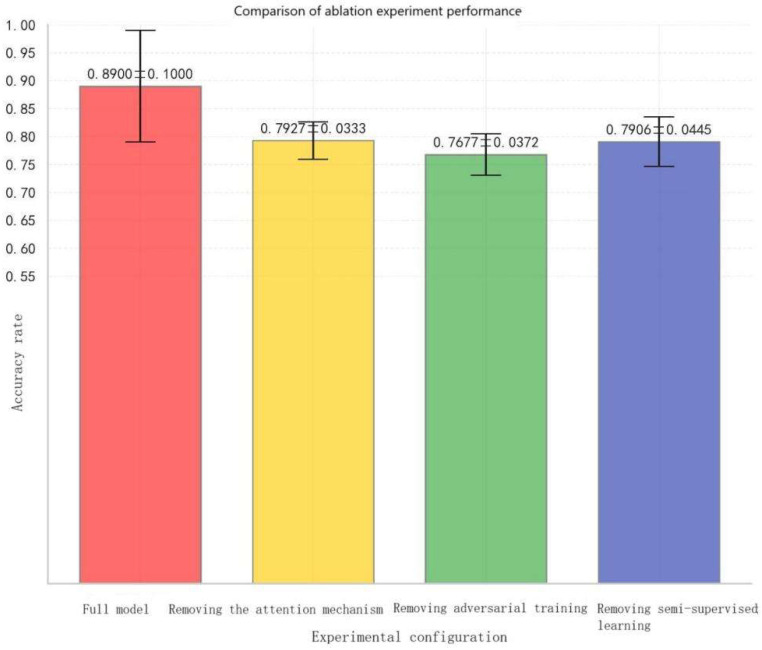
Comparison of ablation experiment performance.

Finally, accuracy was used as the performance metric, and standard deviation was considered to evaluate stability, thereby assessing the effectiveness of the DDPN model in the ablation study. The results are as follows in Table 6:

Figure 14 shows the performance comparison of the ablation study.The results show that the full model achieved the best performance with an accuracy of 0.8900 ± 0.1000. Removing the attention mechanism led to a significant performance drop of 11% (down to 0.7927 ± 0.0333), indicating that the attention mechanism (including both channel and spatial attention) effectively guided the model to focus on critical features, making a substantial contribution to performance improvement. Removing semi-supervised learning resulted in only a slight decrease of 0.0021 in accuracy (to 0.7906 ± 0.0445), suggesting that removing semi-supervised learning (domain discriminator and Gradient Reversal Layer) had a relatively limited impact on model performance and may not play a prominent role in certain task scenarios. In contrast, removing adversarial training further reduced performance by 3% (to 0.7677 ± 0.0372), showing that removing adversarial training had a positive effect on enhancing model performance, though its contribution was weaker than that of the attention mechanism.

Overall, the experimental results demonstrate that module synergy is the core guarantee of model performance. Among them, the attention mechanism played a dominant role, while adversarial training and semi-supervised learning functioned as complementary and auxiliary modules, jointly supporting overall performance.

#### 5.2.3. Comparative Experiments

**Figure 15:**
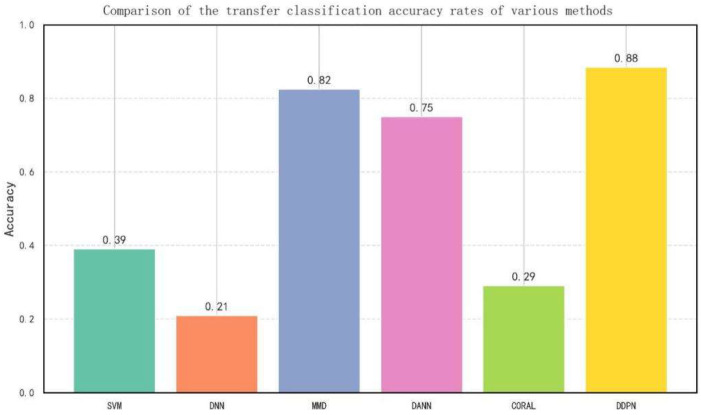
Comparison of the transfer classification accuracy rates of various methods.

To further demonstrate the superiority of the proposed method, we compared it with several classical transfer learning and non-transfer learning approaches, as shown in Figure 15:

SVM: a traditional Support Vector Machine trained on source-domain data.

DNN: a Deep Neural Network trained on source-domain data.

MMD: a deep transfer learning method that achieves domain alignment solely through Maximum Mean Discrepancy (MMD) loss.

DANN: the classical Domain Adversarial Neural Network method.

CORAL: a domain adaptation approach based on covariance matrix alignment.

DDPN (proposed): the complete model incorporating all proposed modules.

The experimental results highlight several key findings. First, the non-transfer learning methods (SVM and DNN), when trained solely on source-domain data and directly applied to the target domain, exhibited poor performance, underscoring the adverse effects of domain shift on traditional models. Second, transfer learning methods such as MMD, DANN, and CORAL achieved considerably better results, demonstrating the effectiveness of transfer learning in mitigating feature distribution discrepancies. Finally, the proposed DDPN model significantly outperformed all comparative methods in both accuracy and F1-score. This superiority is attributed to its multi-stage training strategy and the synergistic integration of attention mechanisms, adversarial training, and semi-supervised learning, which together enable the model to capture cross-domain general features more comprehensively and robustly.

#### 5.2.4. Confusion Matrix and Category-Wise Performance Analysis

**Figure 16:**
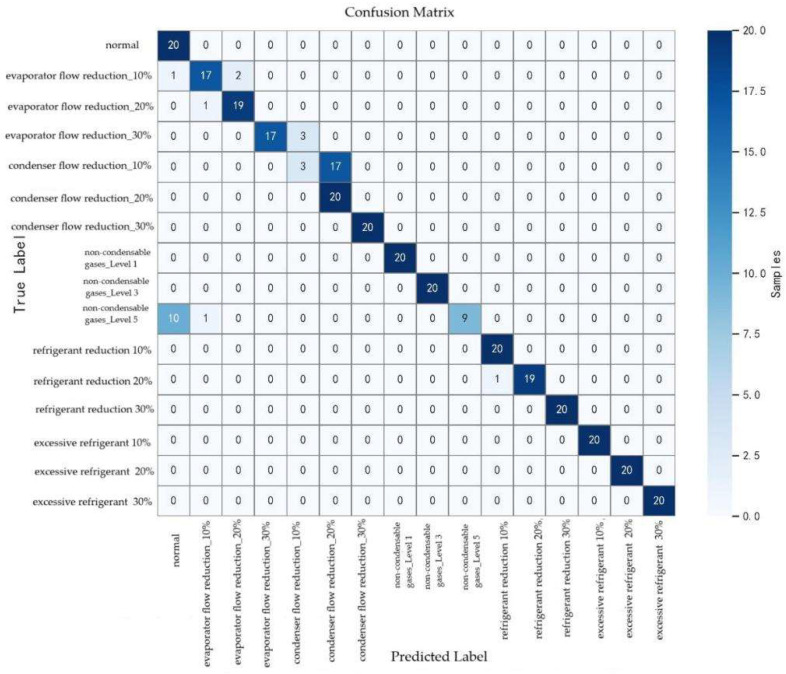
Confusion matrix for classification performance of each fault category.

As shown in Figure 16, the confusion matrix provides a clear view of the model’s classification performance across fault categories. The model achieved an overall accuracy of 89.06% with an F1-score of 0.88, reflecting strong diagnostic capability. For most categories, such as the excessive refrigerant series, non-condensable gases_Level 1, and non-condensable gases_Level 3, the model attained 100% precision and recall, indicating excellent diagnostic reliability.

However, performance varied across certain categories, with noticeable misclassifications. For instance, the recall for condenser flow reduction_10% was only 0.15, with a precision of 0.50 and an F1-score of 0.23. This indicates that most of its 17 samples were misclassified, and half of the predictions assigned to this category were incorrect. Similarly, non-condensable gases_Level 5 had a relatively low recall of 0.40, with 60% of its samples misclassified. The precision for the normal condition was also modest (0.62), suggesting that certain fault samples were mistakenly identified as normal. While the model showed comparatively strong diagnostic performance for evaporator flow reduction, some errors persisted: for example, one evaporator flow reduction_10% sample was misclassified as normal, and three evaporator flow reduction_30% samples were predicted as condenser flow reduction_10%.

These results reveal that the model still struggles to distinguish between fault categories with high similarity, particularly for difficult-to-diagnose cases such as condenser flow reduction_10% and non-condensable gases_Level 5. This underscores the need for further optimization of the model or the development of more discriminative feature representations.

## 6. Conclusions

This paper proposes a heterogeneous transfer learning method that integrates a dual-channel autoencoder, domain adversarial training, and a pseudo-label self-training mechanism. By combining attention mechanisms, adversarial training, semi-supervised learning, and multi-granularity classifiers, the method achieves effective cross-domain knowledge transfer and enhances fault diagnosis performance, enabling accurate few-shot transfer of source domain knowledge to the target domain. Experimental results demonstrate that the proposed DDPN model exhibits high fault diagnosis accuracy in the target domain, effectively identifying common faults in various types of chiller units under conventional operating conditions. In multi-class fault diagnosis tasks, the model achieves higher accuracy and F1-scores compared to traditional methods and existing transfer learning approaches.

Despite these promising results, several directions merit further investigation. First, further exploration of the model’s internal mechanisms is needed to enhance interpretability, enabling engineers to better understand the diagnostic basis. Second, optimizing the architecture and inference speed would facilitate deployment in real-time online fault diagnosis systems. Third, incorporating additional sensor modalities, such as vibration and acoustic signals, could yield a more comprehensive diagnostic framework.

The framework proposed in this study demonstrates commendable performance in addressing heterogeneity among chiller units; however, it is essential to objectively acknowledge its inherent limitations. The method achieves high fault diagnosis accuracy in typical industrial application scenarios, enabling effective identification of common faults in various types of chiller units under conventional operating conditions. It should be noted that performance degradation may occur when the method is applied to units with significant structural disparities compared to the training samples, operating conditions far beyond the training scope, or entirely novel fault patterns not present in the source domain. This limitation primarily stems from two factors: (1) the shared feature space learned through the dual-channel autoencoder and adversarial training may struggle to effectively capture fault features with fundamentally different physical mechanisms; (2) the pseudo-label generation mechanism relies on high-confidence predictions, whose reliability significantly decreases when confronted with previously uncharacterized fault manifestations. Consequently, the effectiveness of this method is contingent upon an important premise: the physical principles underlying faults across different units must possess a certain degree of inherent similarity, even though their surface-level data distributions may exhibit substantial differences. To address this limitation, future research will focus on enhancing the model’s generalization capability under extreme operating conditions, potentially through the incorporation of physics-informed embedding models or few-shot learning techniques, thereby improving its ability to handle edge cases.

We fully acknowledge that the proposed method incurs relatively high computational costs, primarily due to the high computational complexity of the neural network training process. However, it should be noted that the computational demands during the model inference phase are relatively modest. The current research has been conducted primarily on laboratory servers, focusing on principle validation and methodological feasibility demonstration. For subsequent practical shipboard deployment, we suggest optimizing and improving the system through the following two aspects to significantly reduce computational load: adoption of an offline training-online deployment paradigm: the training process will be completed on high-performance servers before deploying the optimized models to practical edge computing platforms; advancement of model lightweighting research: implementation of advanced techniques such as neural network pruning and quantization compression to further enhance operational efficiency.

## Figures and Tables

**Table 1:** Fault List of the Source Domain.

No.	Status	Abbreviation
0	Normal	Normal
1	Reduced condenser water flow rate	FWC
2	Reduced evaporator water flow rate	FWE
3	Excessive refrigerant charge	RO
4	Insufficient refrigerant charge/leakage	RL
5	Presence of non-condensable gases	NC

**Table 2:** Rated Operating Conditions Parameters Table.

Parameters	Values
Chilled Water Inlet Temperature	12 °C
Chilled Water Outlet Temperature	7 °C
Flow Rate	20 m^3^/h
Evaporating Temperature	2 °C
Condensing Temperature	45 °C
Cooling Capacity	90 kW

**Table 3:** Fault List of the Target Domain.

No.	Fault	Fault Induction Method	Diagnostic Model Characteristic Variables
1	Reduced condenser water flow rate	Proportionally reduce the cooling-water flow rate	Evaporation temperature, suction superheat, condensation temperature, evaporator outlet subcooling, discharge temperature, condenser heat-exchange temperature difference, evaporator heat-exchange temperature difference, chilled-water flow rate, cooling-water flow rate, slide-valve position, chiller unit power consumption, etc.
2	Reduced evaporator water flow rate	Proportionally reduce the chilled-water flow rate	
3	Excessive refrigerant charge	Proportionally overcharge refrigerant	
4	Insufficient refrigerant charge/leakage	Proportionally release refrigerant	
5	Presence of non-condensable gases	Proportionally add nitrogen	

**Table 4:** Variables Collected from the Target-Domain Test Bench.

No.	Collected Variables	No.	Collected Variables
1	Compressor suction temperature	13	Chilled-water return temperature
2	Compressor discharge temperature	14	Heat-load inlet water temperature
3	Condenser inlet temperature	15	Heat-load outlet water temperature
4	Condenser outlet temperature	16	Chilled-water flow rate
5	Expansion-valve inlet temperature	17	Chilled-water inlet pressure
6	Expansion-valve outlet temperature	18	Chilled-water outlet pressure
7	Evaporator inlet temperature	19	Cooling-water inlet temperature
8	Evaporator outlet temperature	20	Cooling-water outlet temperature
9	Compressor discharge pressure	21	Cooling-water inlet pressure
10	Condenser inlet pressure	22	Cooling-water outlet pressure
11	Condenser outlet pressure	23	Compressor current
12	Expansion-valve inlet pressure	24	Compressor voltage

**Table 5:** Normal and Fault Operating Data Used for Validation.

Type	Normal	FWC	FWE	RO	RL	NC	Total
Source-Domain Chiller Unit	3393	9376	10,672	10,046	8273	9903	51,663
Target-Domain Chiller Unit	100	300	300	300	300	300	1300

**Table 6:** The effectiveness of the DDPN model in ablation experiments.

Configuration	Accuracy	Accuracy Std	F1
Complete model	0.89	0.1	0.9
Without attention mechanism	0.79	0.033	0.76
Without adversarial training	0.76	0.037	0.73
Without semi-supervised learning	0.79	0.044	0.76

## Data Availability

Data is contained within the article.

## References

[1] Spencer R., Ranathunga S., Boulic M., van Heerden A., Susnjak T. (2025). Transfer learning on transformers for building energy consumption forecasting, A comparative study. Energy Build..

[2] Ren Z., Han H., Cui X., Lu H., Luo M. (2023). Novel data-pulling-based strategy for chiller fault diagnosis in data-scarce scenarios. Energy.

[3] Gao Y., Miyata S., Akashi Y. (2023). Automated fault detection and diagnosis of chiller water plants based on convolutional neural network and knowledge distillation. Build. Environ..

[4] Han H., Ren Z., Cui X., Gu B. (2025). Variable-condition fault diagnosis for building chiller based on deep feature extraction and discrepancy minimization. Energy.

[5] You Y., Tang J., Guo M., Zhao Y., Guo C., Yan K., Yang B. (2024). Ensemble learning based multi-fault diagnosis of air conditioning system. Energy Build..

[6] Xiao F., Zheng C., Wang S. (2011). A fault detection and diagnosis strategy with enhanced sensitivity for centrifugal chillers. Appl. Therm. Eng..

[7] Zhao X., Yang M., Li H. (2012). A virtual condenser fouling sensor for chillers. Energy Build..

[8] Mirnaghi M.S., Haghighat F. (2020). Fault detection and diagnosis of large-scale HVAC systems in buildings using data-driven methods: A comprehensive review. Energy Build..

[9] Gao X., Wu H., Gao H., Qi Y. (2025). Fault Diagnosis of Chiller Units Based on Consistency-Loss Generative Adversarial Network. Chin. J. Sci. Instrum..

[10] Han H., Gao X., Han H., Gao H., Qi Y., Jiang K. (2025). Non-parametric semi-supervised chiller fault diagnosis via variational compressor under severe few labeled samples. Eng. Appl. Artif. Intell..

[11] Gao X.J., Zhang Z.H., Gao H.H., Qi Y.S. (2024). Fault diagnosis of rolling bearings based on a multi-source domain adaptive residual network. J. Vib. Shock..

[12] Gao J., Han H., Ren Z., Fan Y. (2021). Fault diagnosis for building chillers based on data self-production and deep convolutional neural network. J. Build. Eng..

[13] Lu C., Ma X., Yan K. (2024). Chiller fault diagnosis based on improved variational autoencoder and co-training framework: A case study of insufficient samples. J. Build. Eng..

[14] Zhu X., Chen K., Anduv B., Jin X., Du Z. (2021). Transfer learning based methodology for migration and application of fault detection and diagnosis between building chillers for improving energy efficiency. Build. Environ..

[15] Chakrapani G., Sugumaran V. (2023). Transfer learning based fault diagnosis of automobile dry clutch system. Eng. Appl. Artif. Intell..

[16] Li M., Li Y., Li Z. (2025). A comprehensive survey of transfer dictionary learning. Neurocomputing.

[17] Wei J., Chen H., Yuan Y., Huang H., Wen L., Jiao W. (2024). Novel imbalanced multi-class fault diagnosis method using transfer learning and oversampling strategies-based multi-layer support vector machines (ML-SVMs). Appl. Soft Comput..

[18] Na L., Cai B., Zhang C., Liu J., Li Z. (2025). A heterogeneous transfer learning method for fault prediction of railway track circuit. Eng. Appl. Artif. Intell..

[19] Li X., Cheng C., Peng Z. (2025). Hierarchical pseudo-label co-calibration with prototypical decision boundary optimization for mechanical fault diagnosis in label-scarce scenarios. Adv. Eng. Inform..

[20] Wei J., Wang Q., Zhang G., Ma H., Wang Y. (2025). Domain knowledge guided pseudo-label generation framework for semi-supervised domain generalization fault diagnosis. Adv. Eng. Inform..

[21] Chen X., Yin H., Chen Q., Chen L., Shen C. (2024). Multi-source subdomain negative transfer suppression and multiple pseudo-labels guidance alignment: A method for fault diagnosis under cross-working conditions. ISA Trans..

[22] Liu D., Cui L., Wang G., Cheng W. (2024). Interpretable domain adaptation transformer: A transfer learning method for fault diagnosis of rotating machinery. Struct. Health Monit..

[23] Ma J., Lv H., Liu Q., Yan L. (2024). An unsupervised transfer learning gear fault diagnosis method based on parameter-optimized VMD and residual attention networks. J. Braz. Soc. Mech. Sci. Eng..

[24] Song X., Zhu D., Liang P., An L. (2021). A new bearing fault diagnosis method using elastic net transfer learning and LSTM. J. Intell. Fuzzy Syst..

[25] Qian Q., Wen Q., Tang R., Qin Y. (2025). DG-Softmax: A new domain generalization intelligent fault diagnosis method for planetary gearboxes. Reliab. Eng. Syst. Saf..

[26] Zhou J., Qin Y., Chen D., Liu F., Qian Q. (2022). Remaining useful life prediction of bearings by a new reinforced memory GRU network. Adv. Eng. Inform..

[27] Qian Q., Zhang B., Li C., Mao Y., Qin Y. (2025). Federated transfer learning for machinery fault diagnosis: A comprehensive review of technique and application. Mech. Syst. Signal Process..

[28] Zhou J., Yang J., Xiang S., Qin Y. (2025). Remaining useful life prediction methodologies with health indicator dependence for rotating machinery: A comprehensive review. IEEE Trans. Instrum. Meas..

